# Primary hyaline vascular Castleman disease of the kidney: case report and literature review

**DOI:** 10.1186/s13000-019-0870-9

**Published:** 2019-08-22

**Authors:** Yunzhu Li, Haixia Zhao, Bingyin Su, Chan Yang, Shurong Li, Wanlei Fu

**Affiliations:** 1 Department of Pathology, Xinqiao Hospital of Army Medicine University, Chongqing, 400037 China; 2 Development and Regeneration Key Lab of Sichuan Province of Chengdu Medical College, Chengdu, 610500 Sichuan China

**Keywords:** Castleman’s disease, Hyaline-vascular CD, Kidney, Lymphoid node

## Abstract

**Background:**

Castleman’s disease (CD) is an uncommon type of benign proliferation of the lymphoid tissue, characterized by local or systemic lymphadenopathy that most frequently appears in the mediastinum; involvement of the kidney is uncommon, and proliferation originating from the kidney is extremely rare. Herein, we report a rare case of hyaline vascular Castleman’s disease (HV-CD) in a 56-year-old male patient and discuss its morphological characteristics and differential diagnoses including mantle cell lymphoma (MCL), follicular lymphoma (FL), and nodal marginal zone lymphoma (NMZL).

**Case presentation:**

A right upper-middle renal mass was detected after physical examination in a 56-year-old man without any clinical symptoms and a previous partial resection of the right kidney. Microscopically, the lymphoid follicles were increased in number and had expanded mantle zones and atrophic germinal centers. Vascular proliferation and hyalinization in the interfollicular zones were observed. Immunohistochemical staining showed CD20-positive cells in the mantle zones; CD21 and CD35 were expressed in the dendritic cells, CD3 was positive in a small number of T cells, and CD38 and CD138 were positive in the plasma cells. Additionally, Ki-67 expression was positive in the follicle centers. In contrast, staining for Bcl-2 in the germinal centers and cyclin D1 were negative. The immunohistochemical analysis combined with the morphological results supported the diagnosis of HV-CD. The patient recovered well after surgery.

**Conclusions:**

Primarily renal HV-CD without lymph node hyperplasia or clinical symptoms is extraordinarily rare and different from the multicentric-type CD (MCD) with kidney involvement. Therefore, it is extremely important to improve the awareness of this diagnosis. Attention should be paid to the difference between HV-CD and common lymph node reactive hyperplasia, MCL, FL, NMZL, and so on. To avoid misdiagnosis as a renal malignant tumor requiring radical resection, distinguishing these diseases is crucial.

## Background

Castleman’s disease (also known as vascular follicular lymph node hyperplasia, angiofollicular hyperplasia and giant lymph node hyperplasia) is an uncommon type of benign proliferation of the lymphoid tissue; it has been subclassified into five distinct entities on the basis of histologic forms: hyaline vascular CD (HV-CD), plasma cell CD (PC-CD), mixed-type CD, human herpesvirus 8 (HHV- 8)-associated CD and multicentric not otherwise specified CD [1]. Although it can appear in any part of the body, it most frequently appears in the mediastinum [2]. With lymph node enlargement clinically, involvement of the kidney is extremely rare. Only a few cases have been reported worldwide, and few have described the morphological features and differential diagnoses in detail [3–9]. Approximately 80 to 90% of cases of CD are histologically classified as HV-CD, lack systemic symptoms and lack any specificity in laboratory examination results and radiological information, which may lead to misdiagnosis as a renal tumor requiring radical resection. Therefore, histopathology is the unique and gold standard of diagnosis for this lesion, which characteristically manifests as the enlargement of numerous lymphoid follicles with expanded mantle zones and consequent atrophic centers and follicle centers penetrated by increased vessels with fibrous hyalinization [10]. Herein, we report a rare case of HV-CD in a 56-year-old male patient and discuss its morphological characteristics and differential diagnoses.

## Case presentation

A right upper-middle renal mass was detected in a 56-year-old man after a periodic health checkup. His past medical history included hypertension and diabetes, but he was without any clinical symptoms such as fever, nausea, vomiting, frequent urination, urination urgency, or gross hematuria, and without any tenderness in the rib area/rib-waist are. An abdominal computed tomography (CT) scan revealed a node with an isodensity of 4 × 3.5 cm in the upper-middle kidney. There was no other mass on chest X-ray examination, and tumor markers including AFP, CEA, and PSA and laboratory examinations including routine blood and liver function tests were essentially normal except for urinary protein at 3 g/L (reference range: 0–3 g/L) and uric acid slightly increased to 426.1 μmol/L (reference range: 208–428 μmol/L). The patient recovered well after partial resection of the right kidney and tumor by laparoscopy; anti-infective drugs were administered and he was not treated with other adjuvant radiotherapy and chemotherapy.

**Fig. 1:**
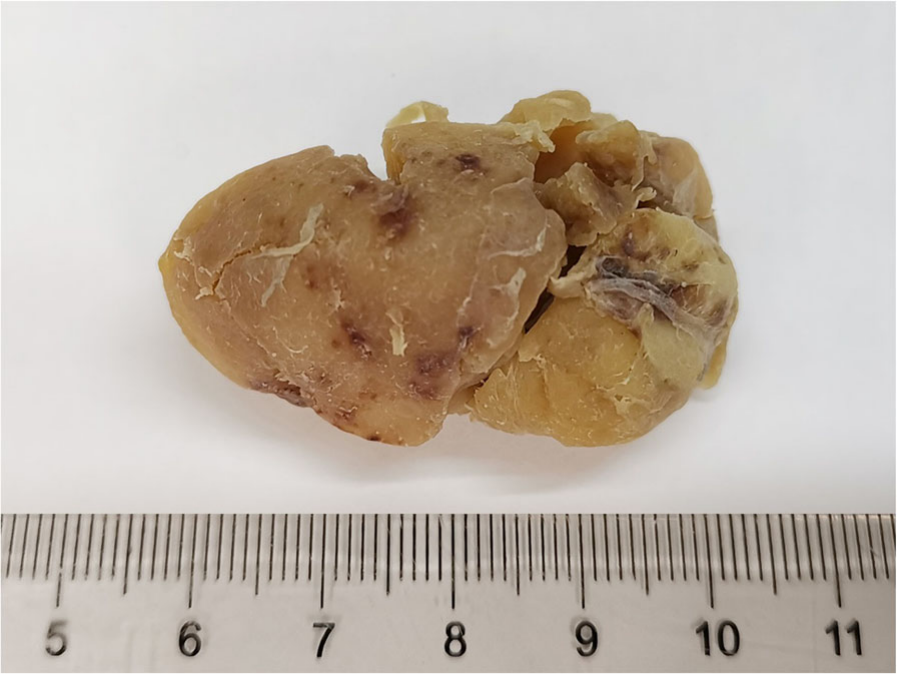
Macroscopic appearance of the kidney mass. The surface of the mass was smooth, and the sectioning showed moderate texture without hemorrhage and necrosis

**Fig. 2:**
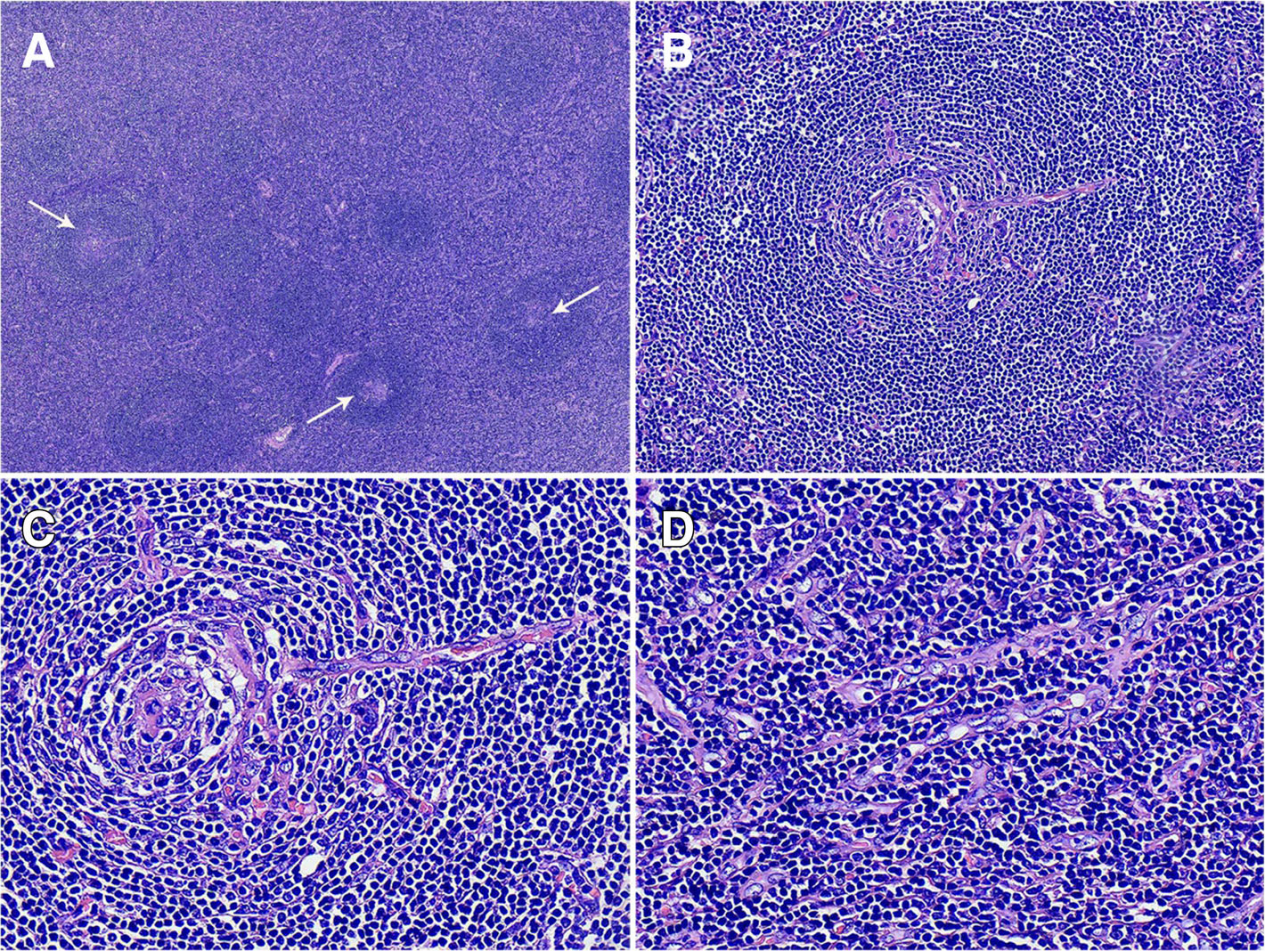
Histological findings of hyaline vascular Castleman’s disease (H-E staining). **a** The lymphoid follicles increased in number and had hypoplastic germinal centers (arrows). **b** The mantle zones were expanded with small lymphocytes with an “onion skin-like” aspect. **c** The follicles were usually penetrated by capillaries and showed a “lollipop” appearance. **d** Strong endothelial venule proliferation and hyalinization in the interfollicular zones was present

On macroscopic examination, the specimen was composed of a nodular mass measuring 4.5 cm × 4.0 cm × 2.0 cm attached to a small portion of the normal kidney cortex (Fig. 1). The surface of the mass was smooth and grayish-brown in color, and sectioning showed moderate texture without hemorrhage and necrosis. The specimen was fixed in a 10% neutral formalin solution, and paraffin-embedded sections were stained with hematoxylin-eosin (H&E) for microscopic examination. Microscopically, the lymphoid follicles were increased in number (Fig. 2a), with an expanded mantle zone of small lymphocytes and atrophic germinal centers, resulting in an “onion skin-like” structure (Fig. 2b). In addition, many of the follicles were penetrated by capillaries and showed a “lollipop” appearance (Fig. 2c). Another characteristic feature is vascular proliferation and hyalinization in the interfollicular zones (Fig. 2d). Follicular dendritic cells and scattered plasma cells were also seen.

**Fig. 3:**
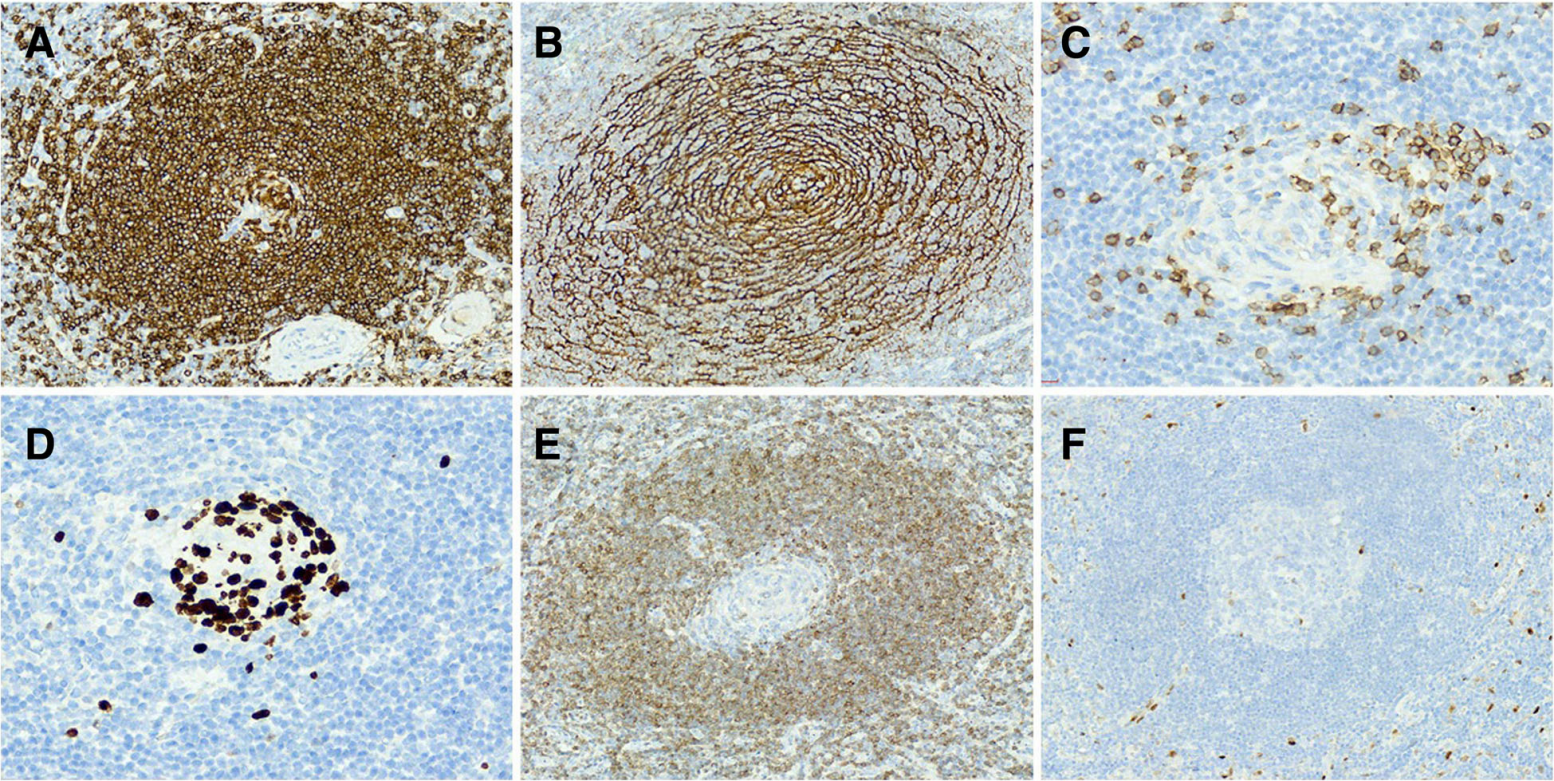
Immunohistochemical findings. **a** The mantle zones consisting of B-cells were positive for CD20. **b** CD21 was present in the concentric follicular dendritic cell networks. **c** The CD3-positive labeled T cells were scattered near the germinal centers. **d** Ki-67 was positively expressed in only the follicle centers. **e** Staining for Bcl-2 was negative in the germinal centers. **f** Cyclin D1 was also negative in HV-CD

Immunohistochemical staining was performed with the EnVision System by a Benchmark-ULTRA automatic immunohistochemical staining instrument (Asia-core, China). The prediluted monoclonal antibodies include Bcl-2, CD20, CD21, CD35, CD38, CD138, cyclinD1, κ light chain, λ light chain, and Ki-67 from the Fuzhou Maxin company. In addition, CD20 showed positive staining in the mantle zones (Fig. 3a), CD21 (Fig. 3b) and CD35 were expressed in the dendritic cells, CD3 was positively scattered among the T cells (Fig. 3c), and CD38 and CD138 were positive in the small plasma cells. Ki-67 expression was positive in the follicle centers (Fig. 3d). In contrast, staining for Bcl-2 in the germinal centers (Fig. 3e) and cyclin D1 (Fig. 3f) were both negative. The staining for κ and λ showed unrestricted light chain expression. In summary, the immunohistochemical analysis supported the diagnosis of HV-CD. Half a year later, the patient recovered well, and there was no recurrence.

## Discussion

Castleman disease is a rare nonneoplastic lymphoproliferative disorder that was first described more than 60 years ago by Castleman as a benign, localized lump or mass [11]. Clinically, the disease has been divided into two subtypes: a unicentric-type CD (UCD) and a multicentric-type CD (MCD). Each of these different subtypes has different clinical symptoms; there is almost no clinical manifestation for UCD. In contrast, MCD includes a plasma cell type and mixed type, which often cause a variety of systemic symptoms, such as fever, emaciation, hypoproteinemia, systemic lymphadenopathy, renal function damage, and even POEMS syndrome (polyneuropathy, organomegaly, endocrinopathy, protein and skin changes) development [12]. The lesions can present anywhere in the body, but are most common in in the mediastinum. Involvement of only the kidney is extremely rare and reported in only a few scattered cases [3–9], in which some patients were misdiagnosed with renal tumors requiring radical resection [8, 9]. The case we described here was the UCD type, presenting with a benign localized lump. There were no clinical signs, and local resection was successful.

Histologically, HV-CD is characterized by broadened mantle zones and atrophic germinal centers, forming an “onion-skin” array. Hyalinized vessel proliferation is associated with endothelial hyperplasia. In contrast to HV-CD, plasma cell-type CD (PC-CD) has its own morphological features. The mantle zones and blood vessels are not hyperplasic in the plasma cells in patients with Castleman’s disease; the morphological features of CD mainly manifest as sheets of mature plasma cell infiltration. Additionally, 50% of cases present monotypic immunoglobulin IgG or IgA λ restriction [13]. CD38- and CD138-positive plasma cells were only scattered in our case. Moreover, the mixed type has the characteristics of both HV-CD and the plasma cell type. HHV-8-related multicentric types mainly occur in HHV-8-positive and other immunosuppressed populations and manifest as a large amount of vascular proliferation between follicles, unclear boundaries between follicles, and immature plasmablasts, which express IgM and λ-restricted light chains and can be potentially labeled with antiviral nuclear antigen LAN-1 [14]. In addition, HHV- 8-negative MCD is similar to the plasma cell variant, but HHV- 8, which frequently occurs in elderly patients, was negative in our patient.

B-ultrasound of HV-CD often shows anechoic space occupying lesions; compared with most tumors, their internal blood flow signal is not strong. There is no obvious specificity in the imaging findings, and it is difficult to distinguish HV-CD from other malignant tumors solely on the basis of imaging findings [15]. Even though the role of FDG-PET/CD is limited, it can be a useful imaging technique in MCD patients and patients with clinical manifestations, such as those associated with UCD [16]. Pathological examination is the primary diagnostic criterion.

In this case, the interfollicular scattering of CD3-positive T cells and positive staining for CD21 and CD35 showed the complete follicular dendritic network (Fig. 2b). Bcl-2 was normally expressed, except in the germinal centers (Fig. 2e). In addition, the CD38/CD138-positive plasma cells showed only scattered expression, different from the slice-like distribution in PC-CD. On the other hand, ki-67 expression was accentuated in the atrophic follicle centers, and there was unrestricted expression in the κ and λ light chains. Based on the above analysis, the mass was suspected to be nonneoplastic in nature, consistent with HV-CD.

From a pathological diagnosis perspective, HV-CD is a diagnosis of exclusion. Before establishing a diagnosis of CD, several lesions with prominent HV-CD-like changes, such as reactive follicular hyperplasia, mantle cell lymphoma (MCL), FL, and NMZL, should be considered. Similar to HV-CD, reactive follicular hyperplasia presents as a benign lesion with different sized follicular “onion-skin” arrays on the mantle rims and “tangible-body” macrophages. However, distinct from HV-CD, the germinal centers are usually expanded, are positive for Bcl-6 B-cells, and show a large amounts of CD57/CD3-positive T cells. However, the absence of Bcl-6 expression and a small amounts of CD57/CD3-positive cells are present in true HV-CD. Another distinction between the two diagnoses is that the follicle dendritic cell network appears tightly in HV-CD, but it is well-defined in hyperplastic reactive lymph nodes [17].

Mantle cell lymphoma (MCL) is a B-cell tumor composed of small- to medium-sized lymphocytes with irregular cleaved nuclei and inconspicuous nucleoli. It is often accompanied by the vitreous degeneration of small blood vessels and can be easily confused with HV-CD. A few cases of MCL with a “mantle zone” growth pattern exhibit considerable histologic overlap with HV-CD, including an “onion skin-like” appearance of mantle cells, an increased number of germinal centers, and proliferated capillaries in the 9 paracortical areas. For example, an extremely rare type of MCL, “in situ” mantle cell lymphoma, is masked by HV-CD-like morphological characteristics, but cyclin D1-positive cells are expressed in the mantle zones of some lymphoid follicles [18]. Pathologists should be aware of this very rare MCL type, and a marker for cyclin D1 or SOX11, which is specifically expressed in MCL but was negative in our case, is especially necessary for HV-CD diagnosis. The t (11, 14) translocation can also be found in most (and possibly all) MCL cases.

Follicular lymphoma (FL) is a germinal center B-cell neoplasm characterized by a nodular growth pattern at low magnification, whose neoplastic follicles are composed of monoclonal B-cells of different proportions and sizes and nonneoplastic cells (follicular dendritic cells and small T cells) [19]. Although the classic manifestations of FL are already recognized by pathologists, one unusual variant of FL, which has a prominent HV-CD-like appearance, including an increased number of follicles, “onion skin-like” mantle zones, and the proliferation of hyalinized blood vessels, requires consideration [20]. Another disease deserving mention is common non-Hodgkin lymphoma (FL), which has its own morphologic features. Neoplastic follicles are more cellular, and the interface between the germinal center and mantle rim is more sharply defined than that in HV-CD. Immunohistochemical staining can be used to distinguish this variant of FL from HV-CD, with positive staining for CD10, Bcl-2 and Bcl-6 in FL but negative staining in HV-CD. In addition, FL also has heavy chain and light chain rearrangements [21] at the t (14; 18)(q32; q21) translocation, which is helpful when morphological and immunohistochemical examinations do not result in a clear diagnosis. Patients with FL often present systemic symptoms and HV-CD-like lesions; the clinical manifestations can help avoid a potential misdiagnosis.

Considering the above HV-CD-like changes, another B-cell lymphoma, nodal marginal zone lymphoma, which is histologically characterized by proliferation of neoplastic B-cells in the marginal zones that infiltrate the atrophic germinal center and surround the remaining lymphoid follicles, should be mentioned. Some reports have demonstrated the difference between NMZL and HV-CD; NMZL presents with clusters of numerous lymphoid cells in the germinal centers, interfollicular areas with substantially broadened large cells, and perifollicular cuffs around the follicular centers that are also expanded by monomorphic lymphocytes [22]. Immunohistochemical analysis revealed that large cells in the germinal centers and lymphocytes in the perifollicular/marginal zone were positive for the pan-B-cell antigens CD20 and CD79a and positive for Bcl-2, also supporting the neoplastic nature of this lymphoma lesion.

The pathogenesis of Castleman’s disease is not clear, but the recognized central factors include immune dysfunction, elevated interleukin-6 (IL-6) levels and HHV-8 infection in MCD have been well demonstrated [23]. The overproduction of IL-6 and upregulation of IL-6 receptor expression can stimulate the proliferation of B lymphocytes, restrain the expression of vascular endothelial growth factor, and result in a series of systemic symptoms such as fever, anemia, hypoproteinemia and proteinuria. Typical therapies targeting IL-6 are effective [24]. In addition, EB virus and HIV infection may also be related to Castleman’s disease, especially MCD [25].

MCD is a systemic disease, but treatment with only surgery is insufficient. Effective treatments for MCD include glucocorticoid control and combination chemotherapy, such as rituximab and monoclonal antibody therapies targeting IL-6. With combination antiretroviral therapy, which is especially necessary, it is easy to cause infection, resulting in poor therapeutic effects [26]. In contrast, in UCD, there are almost no systemic signs, with the exception of a localized benign mass, and the 10-year overall survival rate is more than 95% if complete resection is performed successfully. Surgery is the gold standard for the treatment of unicentric Castleman’s disease [27].

## Conclusion

Renal CD is extremely rare, so it is necessary to improve the awareness of its diagnosis. Because the treatments and prognoses of patients with each type of disease are different, attention should be paid to the differences between HV-CD and common lymph node reactive hyperplasia, mantle cell lymphoma, follicular lymphoma, and nodal marginal zone lymphoma to avoid misdiagnosis as a malignant renal tumor requiring radical resection.

## Data Availability

The data and materials are available to be shared.

## Abbreviations

CD: Castleman disease
HV-CD: Hyaline vascular CD
PC-CD: Plasma cell CD
HHV- 8: Human herpesvirus 8
H&E: Hematoxylin-eosin
CT: computed tomography
UCD: Unicentric type CD
MCD: Multicentric type CD
MCL: Mantle cell lymphoma
FL: Follicular lymphoma
NMZL: Nodal marginal zone lymphoma
